# S119. MULTICULTURAL IDENTITY INTEGRATION AND SCHIZOTYPAL PERSONALITY DISORDER

**DOI:** 10.1093/schbul/sby018.906

**Published:** 2018-04-01

**Authors:** Daisy Lopez, Amy Weisman de Mamani

**Affiliations:** University of Miami

## Abstract

**Background:**

Schizotypal personality disorder (SPD) is often misdiagnosed and understudied. Moreover, when diagnosed correctly, SPD can be difficult to treat and is associated with significant functional impairment. Furthermore, SPD falls under a schizophrenia-spectrum phenotype and can aid in better understanding the trajectory, risk factors, and treatment for psychotic disorders. Given the lack of research on SPD and the underutilization of mental health services by ethnic minorities, this population may be at increased risk for poor outcomes (Delphin-Rittmon, et al., 2015). Yet, few studies assess cultural factors that may account for differences among minorities with psychotic related disorders or SPD. Multicultural identity integration (MII) may offer insights into the presentation of mental illness among ethnic minorities. According to Amoit et al.’s (2007) cognitive-developmental model of social identity configuration, there are four multiple identity configurations. The present study assessed three of the four—categorization, in which individuals identify with one of their cultural groups over others; compartmentalization, in which individuals preserve multiple, separate identities within themselves; and integration, where individuals merge their multiple cultural identities. Research finds that individuals who integrate their culture identities have better mental health outcomes, such as risk for depression, whereas those that do not integrate either culture and compartmentalize their identities, that is, maintain separate identities, have the worst outcomes (Nguyen & Benet-Martinez, 2013). We propose that individuals struggling to integrate identities and instead categorize or compartmentalize them will display higher symptom endorsement of SPD.

**Methods:**

Participants included 261 ethnic minority students from the University of Miami. Students completed measures of schizotypy (Schizotypal Personality Questionnaire; Raine, 1991) and multicultural identities within the self (The Multicultural Identity Integration Scale; Yampolsky et al., 2013). All scales demonstrated good-to-excellent reliability.

**Results:**

When correlating SPD symptoms to the three forms of identity integration, we found a significant correlation with categorization (r =.14, p=.02) and compartmentalization (r =.20, p<.01), however the correlation was non-significant with integration (r =.07, p=.30). When conducting a linear regression using levels of MII to predict SPD, increased levels of categorization (β=.42) and compartmentalization (β=1.53) were associated with greater endorsement of SPD symptoms (F(2,258)=5.49, R2=.04, p<.01).

**Discussion:**

As hypothesized, increased categorization and compartmentalization of multiple cultural identities were associated with greater endorsement of SPD symptoms. Poor adjustment to a new culture and consequential integration of multiple identities may place individuals at risk for developing early symptoms of SPD. However, integration of identities was not significantly related to endorsement of SPD. Therefore, it seems that although poorer integration of identities may serve as a risk factor, greater integration may not necessarily serve as a protective factor. This study is limited by the constricted age range and SES inherent in a college sample. Gathering more information on immigration status, years in the US, etc. may be helpful in highlighting nuances within the data. Interventions targeting individuals with low identity integration may be beneficial to individuals at risk of developing SPD. This is especially true given the real-world functional impairment similar to schizophrenia found among those with SPD.

